# Typical symptoms of common otorhinolaryngological diseases may mask a SARS-CoV-2 infection

**DOI:** 10.1007/s00405-021-06726-4

**Published:** 2021-03-07

**Authors:** Roxanne Weiss, Leon Guchlerner, Andreas G. Loth, Martin Leinung, Sabine Wicker, Volkhard A. J. Kempf, Annemarie Berger, Holger F. Rabenau, Sandra Ciesek, Timo Stöver, Marc Diensthuber

**Affiliations:** 1 Department of Otorhinolaryngology, Head and Neck Surgery, University Hospital Frankfurt, Goethe University, Theodor-Stern-Kai 7, 60590 Frankfurt/M, Germany; 2 Occupational Health Service, University Hospital Frankfurt, Goethe University, Theodor-Stern-Kai 7, 60590 Frankfurt/M, Germany; 3 Institute for Medical Microbiology and Infection Control, University Hospital Frankfurt, Goethe University, Paul-Ehrlich-Str. 40, 60596 Frankfurt/M, Germany; 4 University Center of Competence for Infection Control of the State of Hesse, Goethe University, Paul-Ehrlich-Str. 40, 60596 Frankfurt/M, Germany; 5 Institute of Medical Virology, University Hospital Frankfurt, Goethe University, Paul-Ehrlich-Str. 40, 60596 Frankfurt/M, Germany; 6 German Centre for Infection Research, External Partner Site, Frankfurt, Germany; 7 Fraunhofer Institute for Molecular Biology and Applied Ecology (IME), Branch Translational Medicine and Pharmacology, Frankfurt, Germany

**Keywords:** COVID-19, SARS-CoV-2, Pandemic, Health care workers, Otorhinolaryngological, Medical history

## Abstract

**Purpose:**

Severe acute respiratory syndrome coronavirus type 2 (SARS-CoV-2) replicates predominantly in the upper respiratory tract and is primarily transmitted by droplets and aerosols. Taking the medical history for typical COVID-19 symptoms and PCR-based SARS-CoV-2 testing have become established as screening procedures. The aim of this work was to describe the clinical appearance of SARS-CoV-2-PCR positive patients and to determine the SARS-CoV-2 contact risk for health care workers (HCW).

**Methods:**

The retrospective study included *n* = 2283 SARS-CoV-2 PCR tests from *n* = 1725 patients with otorhinolaryngological (ORL) diseases performed from March to November 2020 prior to inpatient treatment. In addition, demographic data and medical history were assessed.

**Results:**

*n* = 13 PCR tests (0.6%) were positive for SARS-CoV-2 RNA. The positive rate showed a significant increase during the observation period (*p* < 0.01). None of the patients had clinical symptoms that led to a suspected diagnosis of COVID-19 before PCR testing. The patients were either asymptomatic (*n* = 4) or had symptoms that were interpreted as symptoms typical of the ORL disease or secondary diagnoses (*n* = 9).

**Conclusion:**

The identification of SARS-CoV-2-positive patients is a considerable challenge in clinical practice. Our findings illustrate that taking a medical history alone is of limited value and cannot replace molecular SARS-CoV-2 testing, especially for patients with ORL diseases. Our data also demonstrate that there is a high probability of contact with SARS-CoV-2-positive patients in everyday clinical practice, so that the use of personal protective equipment, even in apparently “routine cases”, is highly recommended.

## Introduction

Severe acute respiratory syndrome coronavirus type 2 (SARS-CoV-2) has rapidly spread worldwide since the first documented cases in late December 2019 in China [1]. In Germany, the first SARS-CoV-2-positive case was reported on January 27, 2020 [2] and led to an exponential increase of infections in the following months [3].

The disease caused by SARS-CoV-2, COVID-19 (coronavirus disease 2019), is primarily an acute inflammation of the respiratory system, that can also affect various other organs [4, 5] and in addition could lead to long-term sequelae (‘Long-COVID’) [6]. A characteristic feature of COVID-19, in addition to its high infectivity, is the remarkable variability in the course of the disease, ranging from asymptomatic infections [7, 8] to lethal outcome [4]. In particular, the subgroup of patients who are asymptomatic but still infectious represents a significant risk to spread the infection [8]. This applies especially to otolaryngologists who are exposed to infectious droplets and aerosols during ORL patient examination [9].

Therefore, reliable screening strategies are crucial especially for patients with few or no COVID-19 symptoms. To achieve this goal, a hygiene operational concept was established at our department and has been in use since March 2020 [10]. Part of this concept is a COVID-19 screening by taking a COVID-19-related medical history in combination with a PCR-based SARS-CoV-2 test. For this purpose, a symptom-oriented COVID-19 medical history questionnaire is used in a first step. If this detects symptoms typical of a SARS-CoV-2 infection, the patient is immediately referred to special COVID-19 areas of the hospital (Central Emergency Department or Corona Test Center). Patients with a history considered unremarkable are further examined and treated in the ORL department under strict hygiene precautions. If an inpatient treatment is required, a PCR-based SARS-CoV-2 test is performed prior to admittance, as a second step of the COVID-19 screening concept.

The purpose of this study was to evaluate the rate of positive SARS-CoV-2-PCR screening results in our ORL patient population. In addition, we correlated the symptoms of SARS-CoV-2-positive patients to their ORL disease.

## Patients and methods

### Patients

This study was approved by the local ethics committee (No. 20–1030) and included all patients in the Department of Otorhinolaryngology, Head and Neck Surgery who underwent a throat/nasopharyngeal swab for SARS-CoV-2 PCR screening between March 9, 2020, and November 30, 2020, prior to inpatient treatment. A total of *n* = 2288 SARS-CoV-2 PCR tests were performed in *n* = 1725 patients. 5 tests (0.2%) could not be evaluated due to technical reasons and were therefore repeated. Thus, *n* = 2283 tests were included in this study.

### Medical history questionnaire-based COVID-19 screening

To identify SARS-CoV-2-positive patients before contact with the health care workers (HCW), a COVID-19 medical history questionnaire was created (according to the recommendations of institutional hospital hygiene as specified by the Robert Koch Institute (RKI) and the local public health department). This was given to all patients who presented to our department for examination and treatment since March, 2020. The questionnaire included questions about the typical symptoms of COVID-19 such as (1) fever, (2) cough, (3) changes to smell, (4) changes to taste, (5) shortness of breath, (6) pain in the limbs, (7) sore throat, (8) headache, (9) nausea/vomiting, (10) rhinorrhea, (11) diarrhea, as well as a possible stay outside the country or contact with a confirmed COVID-19 patient (within the last 14 days).

### Molecular biological SARS-CoV-2 screening diagnostics

Laboratory results of a total of *n* = 2283 PCR tests from *n* = 1725 patients were evaluated. For virological diagnostics, respiratory material (throat and nasopharyngeal swabs) was examined by SARS-CoV-2-PCR for the presence of the viral nucleic acid of the novel coronavirus SARS-CoV-2. The following commercially available assays (Table 1) were used in routine diagnostics according to the manufacturers’ protocol: (a) cobas SARS-CoV-2 (Roche Diagnostics International AG, Rotkreuz, Switzerland), (b) Allplex™ 2019-nCoV Assay (Seegene Inc., Seoul, South Korea), (c) Alinity m SARS-CoV-2 AMP Kit (Abbott GmbH, Wiesbaden, Germany), (d) in some cases a rapid PCR-test [Xpert® Xpress SARS-CoV-2 (Cepheid, Sunnyvale, USA)] was used because of a clinical emergency indication (*n* = 127 tests; 5.6% of tests). All (qualitative) PCR assays present the results as cycle threshold (CT) values. Using three quantitative comparison samples containing 10^5^, 10^6^ and 10^7^ SARS-CoV-2 (BetaCoV/Munich/ChVir984/2020) RNA copies/mL a 3-point standard curve was created and viral RNA copies/ml were calculated from the CT values, as described earlier [11]. The comparison samples were obtained from INSTAND e.V. (Düsseldorf, Germany).

**Table 1:** Commercially available SARS-CoV-2-PCR assays which were used in this study

Assay	Target gene(s)	Company	Platform	Method***
cobas SARS-CoV-2	E, ORF1a	Roche diagnostics International AG, Rotkreuz, Switzerland	cobas 6800	NAT
Allplex™ 2019-nCoV Assay*	E, N, RdRP	Seegene Inc., Seoul, South Korea	CFX96™ (Bio-Rad)	NAT
SARS-CoV-2 AMP Kit	N, RdRP**	Abbott GmbH, Wiesbaden, Germany	Alinity m	NAT
Xpert Xpress SARS-CoV-2	E, N2	Cepheid Inc., Sunnyvale, U.S.A	GeneXpert	NAT

*Requires nucleic acid extraction as separate procedure before PCR testing

**Not differentiating between targets

***NAT nucleic acid amplification technique

### Statistics and graphical presentation of the data

All data are given as mean ± standard deviation. A Poisson regression was used for the statistical evaluation of the positive rate of SARS-CoV-2 PCR tests. Statistical analysis was performed with R (version 4.0.2). Graphical presentation of the data was performed using GraphPadPrism 8 (GraphPad Software, San Diego, USA).

## Results

### Patient demographics

The examined patient population (*n* = 1725) consisted of n = 984 male (57.0%) and *n* = 741 female (43.0%) patients. The mean age of the patients was 44.7 (± 24.1) years. Patients were distributed among the following age groups: 0–4 years (*n* = 121; 7.0%), 5–14 years (*n* = 125; 7.2%), 15–34 years (*n* = 383; 22.2%), 35–59 years (*n* = 541; 31.4%), 60–79 years (*n* = 455; 26.4%), and > 80 years (*n* = 100; 5.8%).

### SARS-CoV-2 PCR test results

**Fig. 1:**
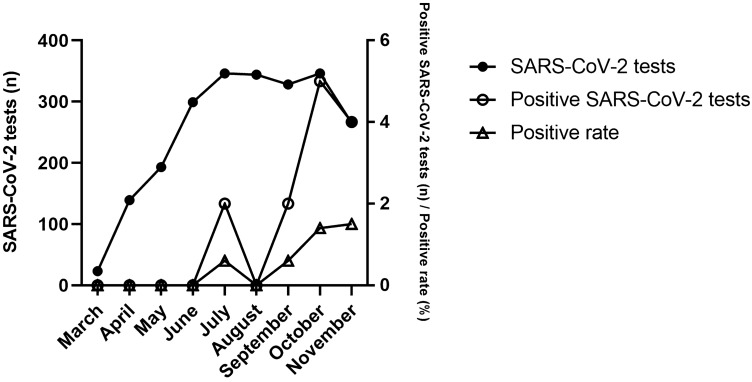
Quantification of SARS-CoV-2 PCR tests (*n* = 2283) performed in patients (*n* = 1725) prior to inpatient admission from March 9 to November 30, 2020. Also shown is the number of positive SARS-CoV-2 PCR tests (*n* = 13 tests from *n* = 13 patients). A significant increase in the positive rate during the observation period was found (*p* < 0.01)

An average of 8.6 ± 5.5 PCR tests was performed per day during a time period of 267 days for ORL patients (*n* = 2283 tests in total). The number of PCR tests performed per day ranged from *n* = 0 to *n* = 21. The monthly PCR test rate was 253.7 ± 113.4 tests and varied from *n* = 23 tests in March to n = 346 tests in July and October 2020. A positive result was found in *n* = 13 PCR tests from *n* = 13 patients (positive rate of PCR tests performed 0.6%), indicating that 0.8% of the tested patients were SARS-CoV-2 positive. The first two positive cases (15.4%) occurred in July, and a total of 11 cases (84.6%) were identified in September, October and November. The monthly positive rate of SARS-CoV-2 PCR tests varied from 0% (March to June, August) to 1.5% in November and increased significantly from March to November 2020 (*p* < 0.01). The results of the PCR tests for SARS-CoV-2 are shown in Fig. 1.

In the PCR tests positive for SARS-CoV-2 (*n* = 13) the detected viral load ranged from 100 to 10.5 × 10^6^ RNA copies/mL. Overall, four patients (30.8%) had a viral load > 10^6^ RNA copies/mL and were thus categorized as highly infectious.

### SARS-CoV-2-positive patients

All patients with a positive SARS-CoV-2 PCR test were adults (*n* = 13). The mean age of these patients was 42.5 ± 14.1 years. The youngest patient was 19.5 years old and the oldest patient was 67.9 years old. Seven patients (53.8%) were female and six were male (46.2%). Six patients with a positive PCR test (46.2%) presented as emergency cases. Seven patients tested positive (53.8%) were scheduled for elective inpatient treatment. Four patients (30.8%) presented to our department due to an otologic disease (cholesteatoma, traumatic perforation of the tympanic membrane, otosclerosis, cephalgia after cochlear implant surgery), two patients (15.4%) had a rhinologic disease (chronic rhinosinusitis, nasal bone fracture), and seven patients (53.8%) had a disease of the neck area (tongue base hyperplasia, reduced general condition after radiochemotherapy of a laryngeal carcinoma, peritonsillar abscess, tongue base tonsillitis, laryngeal carcinoma, acute laryngopharyngitis, chronic tonsillitis). The demographic data of SARS-CoV-2-positive patients are shown in Table 2.

**Table 2:** Demographic data of the SARS-CoV-2 positive patients (*n *= 13). *SD* standard deviation

	Patients (*n*)	Ratio (%)
Total	13	100
*Gender*		
Male	6	46.2
Female	7	53.8
Age, mean (± SD)	42.5 (± 14.1)	
0–4	0	0.0
5–14	0	0.0
15–34	5	38.5
35–59	7	53.8
60–79	1	7.7
80 +	0	0.0

### COVID-19 medical history questionnaire results in SARS-CoV-2-positive patients

The data on the clinical symptoms of the SARS-CoV-2-positive patients (*n* = 13) are summarized in Tables 3 and 4.

**Table 3:** Frequency of symptoms of the SARS-CoV-2 positive patients (*n* = 13)

	Patients (*n*)	Ratio (%)
*Symptoms*		
Fever	2	15.4
Cough	2	15.4
Changes to smell	2	15.4
Changes to taste	3	23.1
Shortness of breath	2	15.4
Pain in the limbs	0	0.0
Sore throat	3	23.1
Headache	5	38.5
Nausea/vomiting	1	7.7
Rhinorrhea	1	7.7
Diarrhea	1	7.7
*Number of symptoms*		
0	4	30.8
1	1	7.7
2	5	38.5
3	1	7.7
4	2	15.4
> 4	0	0.0

Data were obtained from the COVID-19 medical history questionnaires

**Table 4:** Viral load of the SARS-CoV-2 positive patients (*n* = 13), the symptoms reported in the COVID-19 medical history questionnaire as well as the ORL diagnosis or secondary diagnosis by which COVID-19 was masked

Patient	Otorhinolaryngological diagnosis/ secondary diagnosis	Symptom (s)	Viral load (RNA copies/mL)	
			< 10^6^	> 10^6^
1	Superinfected cholesteatoma	Headache	X	
2	Reflux disease Tongue base hyperplasia	Sore throat		X
	Arterial hypertension	Headache		
3	Reduced general condition after primary radiochemotherapy for laryngeal carcinoma	Shortness of breath Fever		X
4	S/P paranasal sinus surgery	Changes to smell		X
	Bronchial asthma	Cough		
5	Chronic rhinosinusitis with nasal polyps	Changes to taste Rhinorrhea	X	
6	Peritonsillar abscess	Sore throat Headache Changes to taste	X	
7	S/P cochlear implantation Migraine	Headache	X	
	Familial mediterranean fever	Cough Shortness of breath		
	Drug side effect (Tocilizumab, Colchicin)	Nausea/vomiting		
8	Tongue base tonsillitis	Fever Sore throat Headache		X
	Drug side effect (antibiotics)	Diarrhea		
9	Traumatic perforation of the tympanic membrane with involvement of annulus fibrosus tympani (possible lesion of the chorda tympani)	Changes to smell Changes to taste	X	
10	Laryngeal carcinoma	None	X	
11	Acute laryngopharyngitis	None	X	
12	Chronic tonsillitis	None	X	
13	Nasal bone fracture	None	X	

Four patients did not report any of the symptoms listed in the questionnaire that are typical of COVID-19

*S/P* status/post, *RNA* ribonucleic acid

Nine patients (69.2%) confirmed the presence of at least one of the listed 11 symptoms. Overall, a wide range of symptoms was reported: 10 of the 11 symptoms queried were confirmed by at least one patient. None of the patients had more than four symptoms. At least one of the most common COVID-19 symptoms, i.e. cough, fever and shortness of breath [4, 12], was present in four patients (30.8%). Four patients (30.8%) reported no symptoms at all. No patient (0.0%) reported having been abroad recently. Two patients (15.4%) reported direct contact to a confirmed COVID-19 patient within the past 14 days.

## Discussion

From a very early stage of the COVID-19 pandemic, evidence was accumulating that the upper respiratory tract is the main reservoir for SARS-CoV-2 [13]. Several medical societies, such as the German Society of Otolaryngology, Head and Neck Surgery [14] or the American Academy of Otolaryngology—Head and Neck Surgery [9] pointed out early that otolaryngologists are at high risk for infection. Numerous studies suggest that transmission of SARS-CoV-2 occurs primarily through droplets and aerosols [15–18]. Surgical procedures in the upper airways are, therefore, considered to be potential high-risk procedures as they are associated with aerosol and droplet exposure, especially when active instruments are used [19, 20]. Even standard ORL examination with nasal endoscopy has to be regarded as a potentially droplet- and aerosol-generating intervention with a considerable risk of virus transmission [21]. Moreover, a prospective cohort study has recently shown that HCW have a more than sevenfold increased risk for a severe course of COVID-19 in case of infection [22].

Early identification of infectious patients is, therefore, crucial to reduce both the uncontrolled spread of the pandemic and, in particular, the risk of patient-to-HCW transmission. Usually, COVID-19 manifests itself with symptoms such as cough, fever and shortness of breath [4]. Therefore, a COVID-19 medical history questionnaire has been used at our department since March 2020 to detect symptoms typical of COVID-19. The main purpose of this procedure was to identify clinical cases suspicious for COVID-19 before they had contact to HCW and other patients to avoid nosocomial transmissions. Patients with an unremarkable medical history who were to undergo inpatient treatment in addition received a PCR-based SARS-CoV-2 test on admittance.

Data collected in this study show that the number of tests performed increased from March to June 2020. This increase is mainly explained by the fact that in the first months of the pandemic, inpatient treatment at our hospital was limited only to emergency and oncology cases. The number of PCR tests performed, therefore, corresponds to the clinically necessary inpatient admissions of patients and was not limited by the availability of the PCR test itself. The discrepancy between the number of PCR tests performed (*n* = 2283) and the number of patients examined (*n* = 1725) resulted from multiple testing of individual patients (e.g., due to routine re-screening after 7 days of hospitalization or repeated inpatient admission).

After a plateau phase in the number of PCR tests performed in the months of July to October 2020, a reduction of inpatient capacity and elective procedures was again required from the end of October 2020 due to the second wave of the COVID-19 pandemic. This led to fewer SARS-CoV-2 PCR tests being performed. However, despite reduced numbers of PCR tests, there was a substantial increase of positive SARS-CoV-2 test results in this period. Consequently, the positive rate of SARS-CoV-2 PCR tests increased significantly during this period up to 1.5% in November 2020 (*p* < 0.01).

It is remarkable that no patient was tested positive for SARS-CoV-2 from March to June (first wave of the pandemic). A limited availability of SARS-CoV-2 tests in the early phase of the pandemic cannot be the reason for this finding because all patients were tested prior to hospitalization during this period as well (March to June 2020: *n* = 654 PCR tests performed). Thus, in these months together, more SARS-CoV-2 tests were performed than in the following period from October to November (*n* = 611 PCR tests in total). However, there were nine SARS-CoV-2-positive patients from October to November 2020. This represents nearly 70% of all patients who were tested positive during the entire observation period. Although the number of SARS-CoV-2-positive patients in this study is small, our data suggest that the second wave of the pandemic beginning in the end of September 2020 had a much larger scale than the first wave in spring of 2020.

To frame this result, it is helpful to consider the epidemiological data of the federal state our hospital is located in. They also show a sharp increase in positive SARS-CoV-2 test results for October and November 2020 [23]. The SARS-CoV-2 screening results obtained on our patient group, therefore, seem to reflect the development of the incidence of infections at the federal state level. Our findings may, therefore, suggest a possible future use of routine PCR-based SARS-CoV-2 screenings in ORL clinics as part of an infection monitoring or even a national surveillance strategy.

When looking at the age distribution of patients with positive test results, it is noticeable that 12 of 13 SARS-CoV-2 positive cases (92.3%) are found within the age group of 15–59 years (19.5–59.3 years). This indicates a clustering of cases in this age group, similar to the state [23] and national [3] levels. However, the age distribution of our patient group is only comparable to a limited extent to that of COVID-19 cases in the general population. This is due to the small number of examined cases and a pre-selected composition of the studied group of patients (ORL patients). This limitation is also reflected by the fact that no children were among our SARS-CoV-2-PCR-positive patients.

Molecular SARS-CoV-2 diagnostics (PCR tests) revealed the detection of viral RNA in 13 patients. Almost one-third of the SARS-CoV-2-PCR-positive individuals in our patient group (*n* = 4) had a viral load > 10^6^ RNA copies/mL, indicating high infectivity. A major finding of this work is that none of these 13 SARS-CoV-2-PCR-positive patients was identified as a likely COVID-19 case by the questionnaire-based medical history.

In fact, approximately one-third of the SARS-CoV-2-positive patients (*n* = 4; viral load < 10^6^ RNA copies/mL in each case) reported no clinical symptoms at all. It is known that a SARS-CoV-2 infection is not always associated with symptoms. Nearly 20% of all patients with a SARS-CoV2 infection experience an asymptomatic course of the disease [24]. In addition, the group of presymptomatic patients who do not yet report symptoms at the time of SARS-CoV-2 detection must be taken into account as they may not develop symptoms until subsequent days. Patients without symptoms are not identifiable as COVID-19 cases by their medical history, but must be considered infectious at this time [8].

In all SARS-CoV-2-PCR-positive patients with symptoms (*n* = 9), only a few symptoms (four or fewer of the 11 queried symptoms) were present. The symptoms reported by the patients, included cough, fever, shortness of breath, and changes to smell/taste, which are among the most common manifestations of COVID-19 [4, 12, 25]. Thus, clinical characteristics of a SARS-CoV-2 infection were present in the majority of the patients. According to the medical assessment, however, all reported symptoms were attributable to the existing ORL diseases or secondary diagnoses of the patients. None of these patients were immediately suspected of suffering from COVID-19. Various studies have shown that COVID-19 frequently manifests with symptoms of the ORL region [26]. The findings of our work point to a possibly resulting diagnostic dilemma. The typical symptoms associated with the ORL disease may be indistinguishable from COVID-19 symptoms. Most of the symptoms typical for COVID-19 are non-specific, but at the same time represent common symptoms of ORL diseases. This leads to an uncertain clinical and diagnostic situation, since typical symptoms of ORL diseases can ‘mask’ the symptoms of COVID-19. Thus, our results demonstrate that medical history alone is of limited help to rule out a SARS-CoV-2 infection in ORL patients and PCR testing is therefore strongly encouraged.

## Limitations of the study

Limitations of our study result from the small number of COVID-19 cases and the inhomogeneous age distribution of the patient group. There may be also a selection bias of the patients studied, since only inpatients without a medical history typical for a SARS-CoV-2 infection were included in this study. Patients with a history typical of COVID-19 were referred to the specialized COVID-19 areas of the hospital. Furthermore, the patient group included in this study comprises only patients who were scheduled for inpatient admission. Outpatients did not undergo PCR testing for SARS-CoV-2 infection and were therefore not included into this study.

Another limitation may also be related to a falsification of the data by the patients themselves. The symptom-oriented COVID-19 medical history questionnaire used was completed by the patients (or by their parents in the case of children). Since all patients required an inpatient treatment of their disease, it cannot be excluded that patients may have unconsciously or even consciously given inaccurate information about the presence of symptoms typical for COVID-19 to gain unlimited medical care. This assumption is supported by the fact that four SARS-CoV-2-positive patients did not report any symptoms in the COVID-19 medical history questionnaire. Even with an asymptomatic COVID-19 disease at least some symptoms should have been mentioned relating to the present ORL disease. However, even if this assumption was true, it would not change the overall conclusion that a subjective medical history is of limited value compared to an objective PCR test result.

Finally, the studied patient cohort is not representative of the general population in terms of age distribution. Even though a number of known and unknown influencing factors affected the composition of the studied patient cohort, it represents the clinical routine of a university ORL clinic. In this respect, the results do not represent the incidence of SARS-CoV-2 infections in the general population, but in the ORL patient population during the COVID-19 pandemic between March and November 2020.

## Conclusions

Our data confirms an increase in the number of COVID-19 cases in the fall of 2020. A relevant proportion of patients reported no symptoms, although SARS-CoV-2 was detectable in the throat/nasopharyngeal swab using a PCR test and the patients therefore had to be regarded as infectious. Since numerous ORL diseases are associated with COVID-19-like symptoms, it has to be assumed that in a relevant proportion of patients with ORL diseases there is a ‘masking’ of the SARS-CoV-2 infection by the ORL disease-typical symptoms. Although only inpatients with no or minor symptoms were examined in this study, it is most likely that the rate of SARS-CoV-2-positive cases within the ORL outpatient population is likely to be similar. Our data demonstrates a probability of an unrecognized COVID-19 patient contact for an otolaryngologist of approximately 0.6% (i.e., about one in 200 physician–patient contacts). Accordingly, taking a medical history alone for screening purposes cannot replace SARS-CoV-2 laboratory diagnostics. Adequate personal protective equipment that reliably prevents infection of HCW is, therefore, strongly recommended for all ORL patient examinations.
